# Supplementary material for: Sulforaphane-Induced Cell Mitotic Delay and Inhibited Cell Proliferation via Regulating CDK5R1 Upregulation in Breast Cancer Cell Lines
Source: Biomedicines. 2023 Mar 23;11(4):996. doi: 10.3390/biomedicines11040996 (PMC10135833; doi:10.3390/biomedicines11040996)
Supplement: Supplementary file 1 [file biomedicines-11-00996-s001.zip › biomedicines-2224129-SI.pdf]

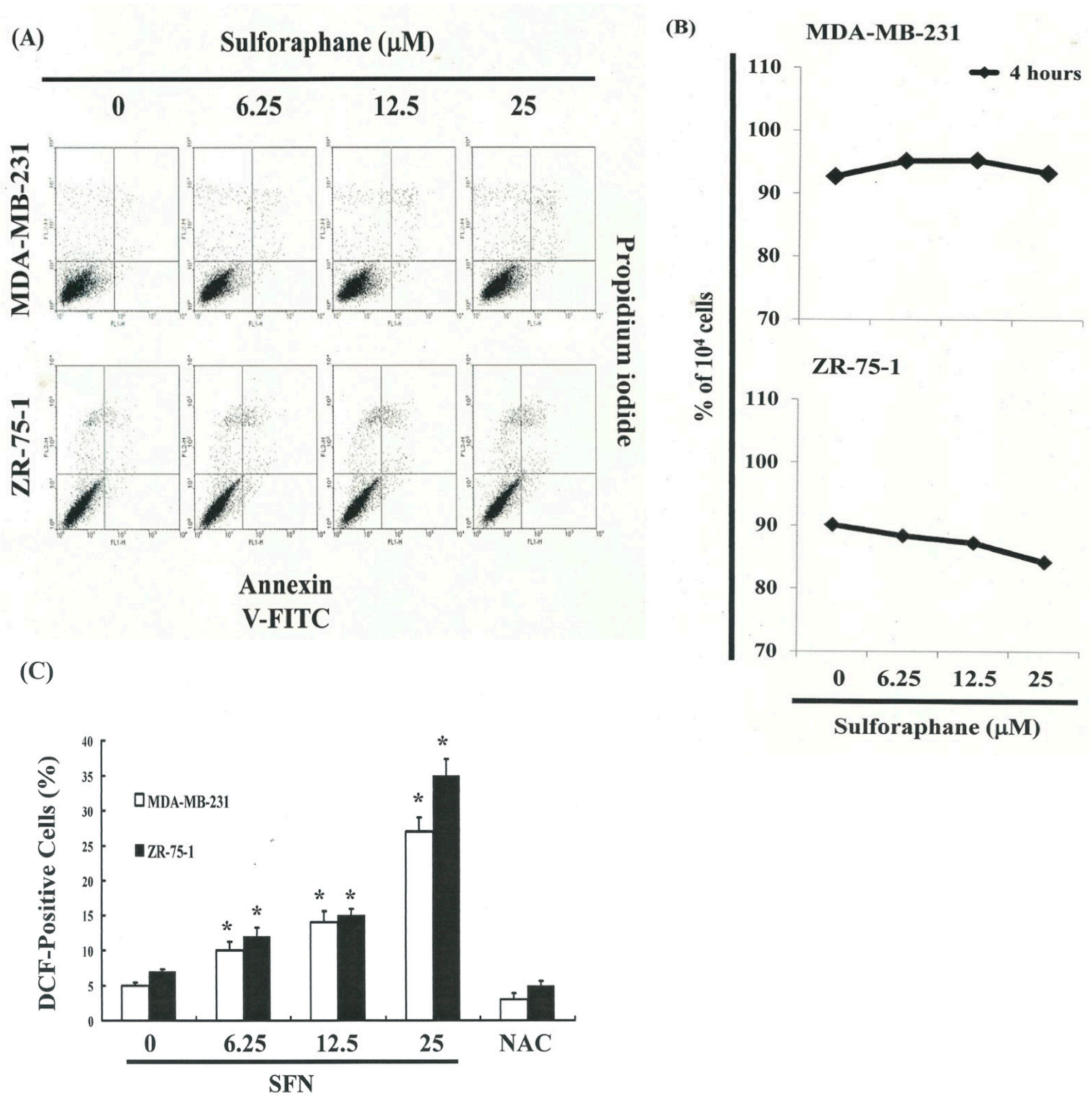

Figure S1. The effect of SFN on apoptosis/necrosis in the MDA-MB-231 and ZR-75-1 cells.

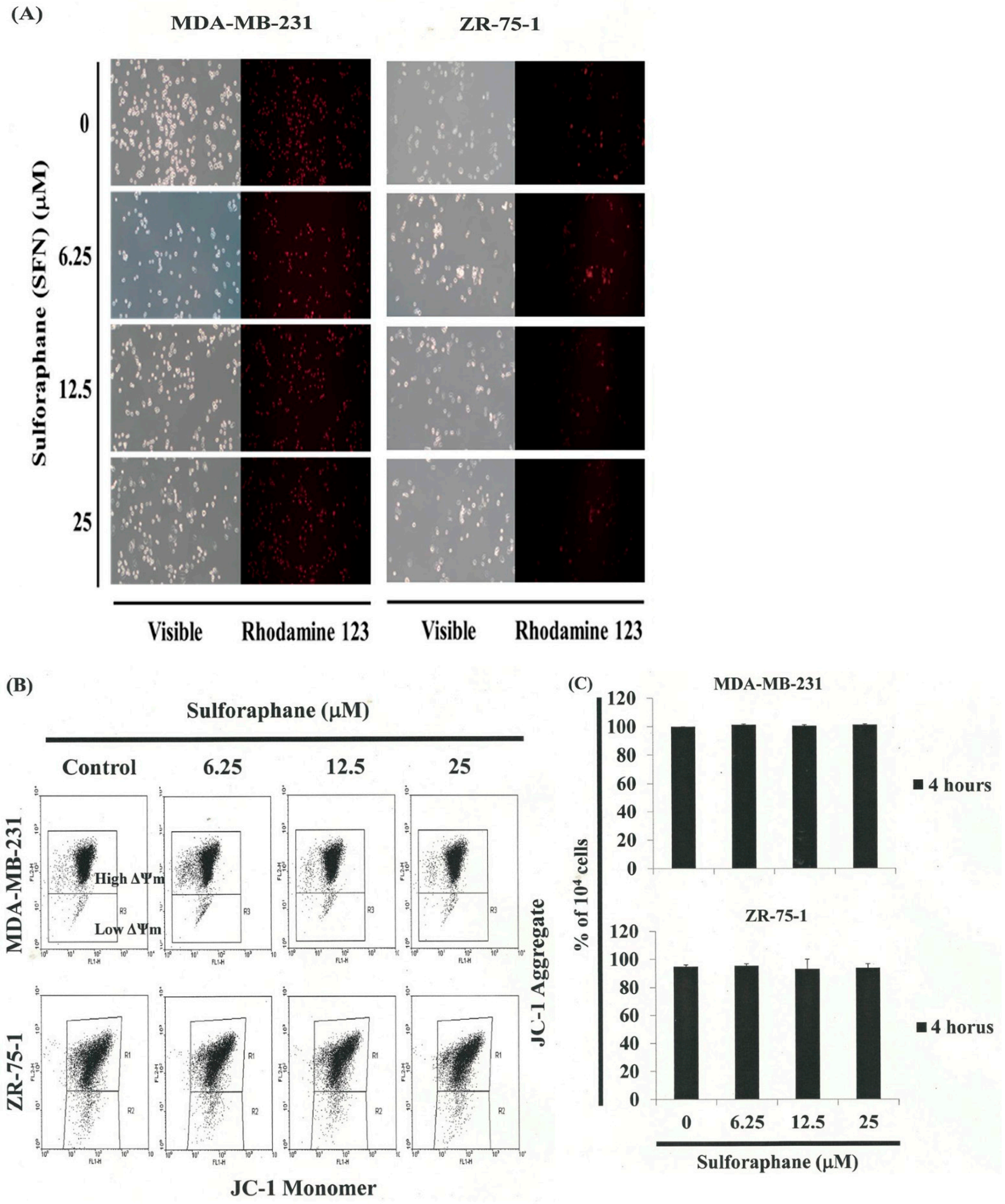

Figure S2. SFN mediated mitochondrial membrane potential (ΔΨm)-independent anti-proliferation in MDA-MB-231 and ZR-75-1 cells.

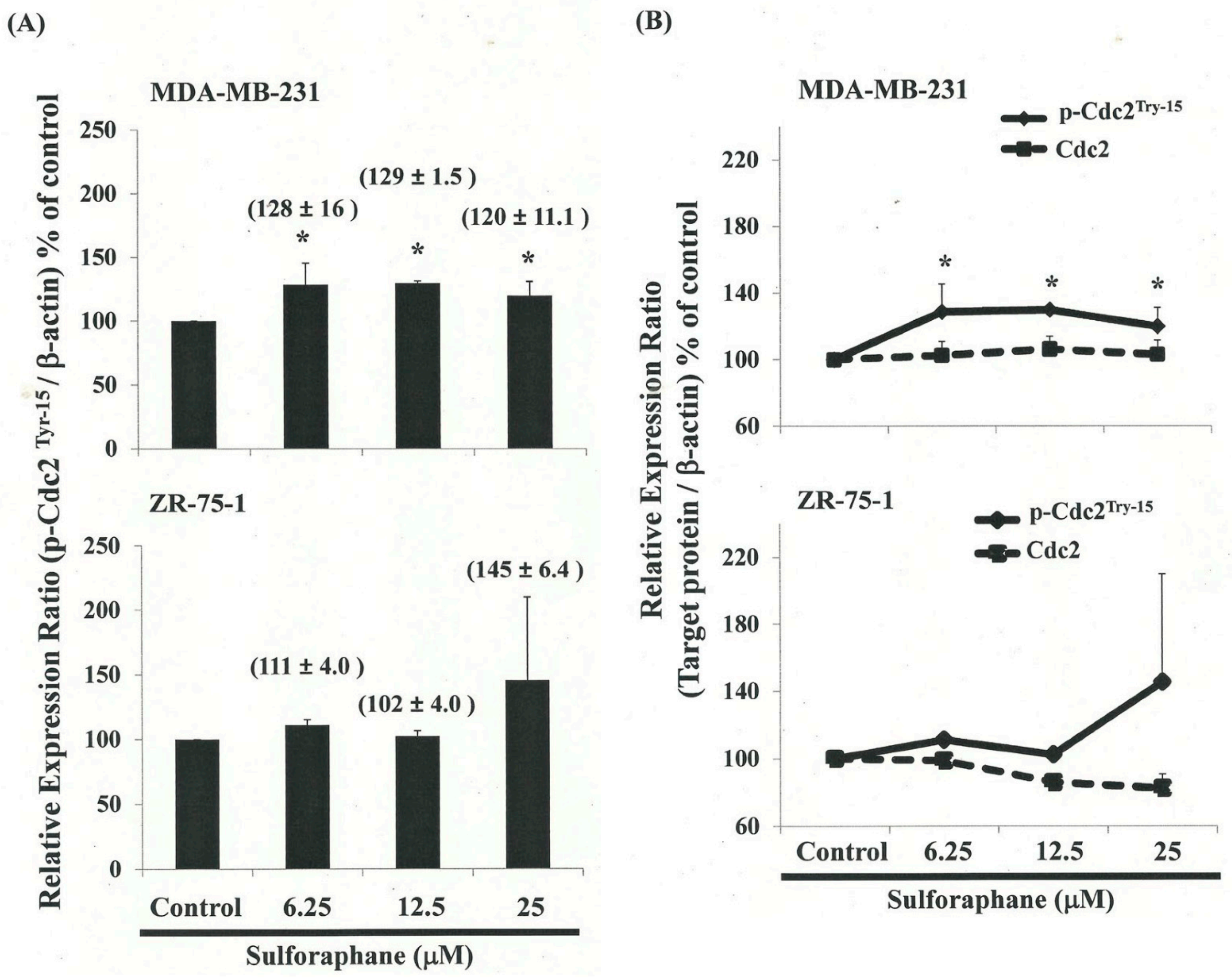

Figure S3. SFN repressed CDC2, CDC25C activity in MDA-MB-231 and ZR-75-1 cells.

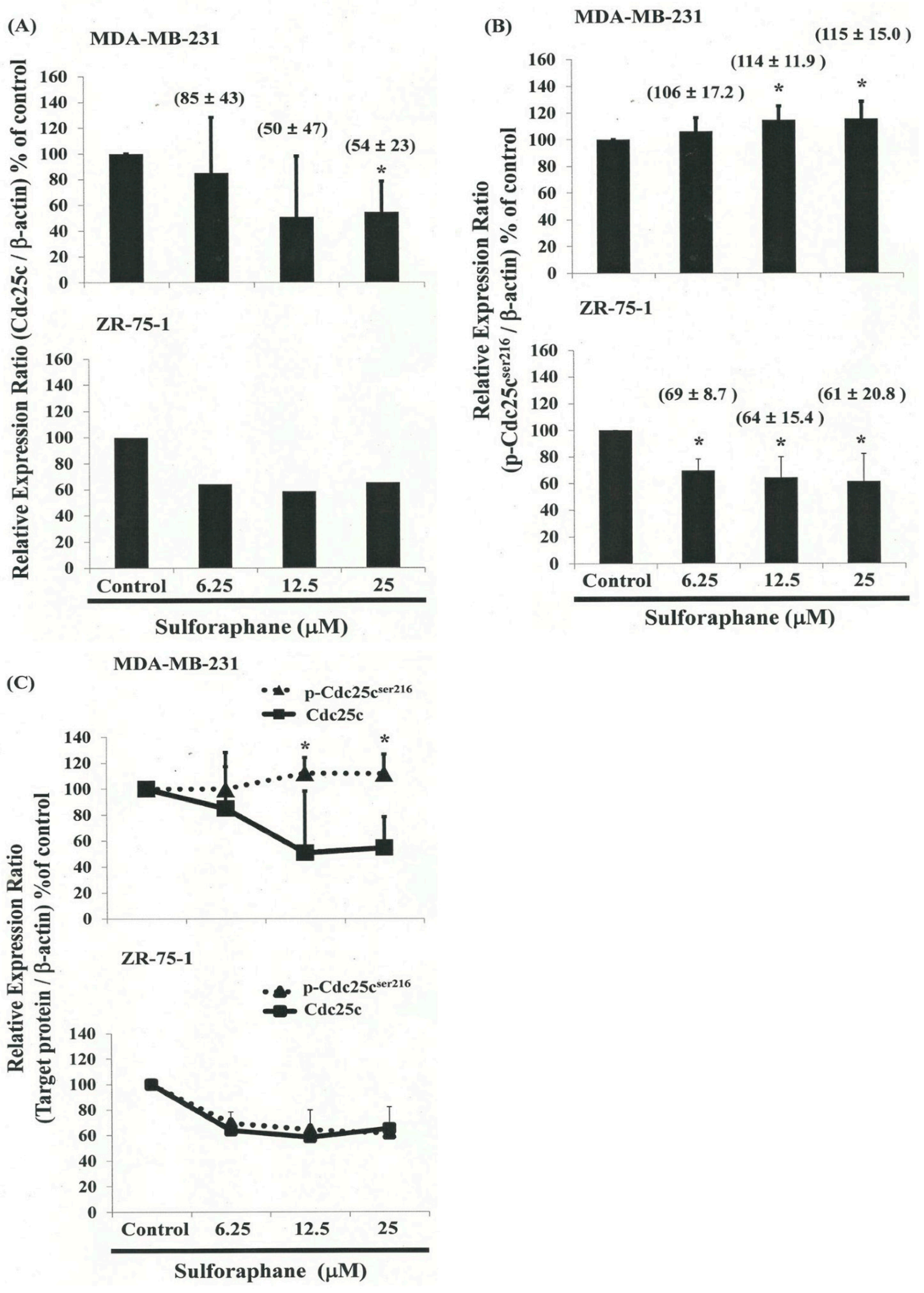

Figure S4. Protein expression in breast cancer cells (A) cdc25c (B) p-cdc25c (C) p-cdc25c:cdc25c.

Table S1

Human Cell Cycle PCR Array pathways of the genes up- or down-regulated in MDA-MB-231 cells following exposure to SFN (25 μM)

Rank	Pathway
1	G1 Phase and G1/S Transition
2	S Phase and DNA Replication
3	G2 Phase and G2/M Transition
4	M Phase
5	Cell Cycle Checkpoint and Cell Cycle Arrest
6	Regulation of Cell Cycle
7	Negative Regulation of Cell Cycle

Table S1. Human cell cycle PCR array pathways of the genes up- or down- regulated in MDA-MB-231 cells following exposure to SFN (25μM).

Table S2

The genes up-regulated in MDA-MB-231 cells following exposure to SFN (25 μM)

Gene	Fold change	Description
BIRC5	4.2	Baculoviral IAP repeat containing 5
CCND1	5.3	Cyclin D1
CDC34	7.7	Cell division cycle 34 homolog (S. cerevisiae)
* CDK5R1	24.7	Cyclin-dependent kinase 5, regulatory subunit 1 (p35)
E2F4	7.4	E2F transcription factor 4, p107/p130-binding
MCM5	5.6	Minichromosome maintenance complex component 5
TP53	4.9	Tumor protein p53

Table S2. The genes up-regulated in MDA-MB-231 cells following exposure to SFN (25μM).
